# Perceived Challenges and Solutions to Adopting Healthy Diets Among Women and Children: A Photovoice Study in Urban Ethiopia

**DOI:** 10.1111/mcn.70208

**Published:** 2026-06-17

**Authors:** Meron Worku, Michelle Holdsworth, Marie Ruel, Ana Irache, Kaleab Baye, Mark Spires, Nicolas Bricas, Rebecca Pradeilles

**Affiliations:** ^1^ College of Natural and Computational Sciences, Center for Food Science and Nutrition Addis Ababa University Addis Ababa Ethiopia; ^2^ Menelik II Medical and Health Science College Addis Ababa Ethiopia; ^3^ UMR MoISA (Montpellier Interdisciplinary Centre On Sustainable Agri‐Food Systems) Univ Montpellier, CIRAD, CIHEAM‐IAMM, INRAE, Institut Agro, IRD Montpellier France; ^4^ Nutrition, Diets & Health Unit (NDH) International Food Policy Research Institute Washington DC US; ^5^ Nursing Department, Faculty of Medicine Universidad Autónoma de Madrid Madrid Spain; ^6^ Nutrition and Food Systems Division Research Center for Inclusive Development in Africa (RIDA) Addis Ababa Ethiopia; ^7^ Centre for Food Systems Research, Natural Resources Institute University of Greenwich London UK

**Keywords:** children under five, double burden of malnutrition, food environment, healthy diets, photovoice, urban Ethiopia, women of reproductive age

## Abstract

Ethiopia has one of the highest rates of undernutrition among children under five (U5) and women of reproductive age (WRA) globally, alongside rising overweight/obesity, particularly in urban areas. Poor diet is a shared driver of multiple forms of malnutrition. We used a participatory photography (Photovoice) approach to explore the lived experiences of WRA and their children U5 in adopting healthy diets across lower‐ and higher‐ socio‐economic status (SES) groups in Addis Ababa. Women took photographs illustrating challenges to healthy diets, and five focus groups (*n* = 31 women) were conducted to discuss challenges and solutions, with separate sessions held for different SES groups. A hybrid thematic analysis, combining deductive and inductive approaches, identified themes/subthemes, with comparisons across SES groups. Financial and physical barriers to accessing healthy foods, time constraints and perceived poor food safety were major contributors to poor diets. In lower SES groups, women also reported limited knowledge about healthy diets, inadequate family support and poor home food environments. In higher SES groups, unhealthy food preferences coupled with easy access to and aggressive promotion of unhealthy foods were key challenges. Proposed government‐level solutions included job creation, nutrition education, affordable healthy food, investment in household infrastructure, expanded childcare and restrictions on unhealthy food availability and promotion. Societal‐level solutions included gender equality, strengthened community‐based loan schemes and support for urban agriculture. These findings highlight that women recognise their needs and who should support them, and emphasise the importance of including women's voices in decision‐making processes. Findings also underscore the need for integrated interventions targeting individual, food environment and socio‐economic drivers to improve diets among women and children in urban Ethiopia.

## Summary

- Time constraints, financial and physical inaccessibility and poor food safety are perceived as major barriers to adopting a healthy diet across socio‐economic groups.
- Inadequate household infrastructure in deprived areas hinders healthy meal preparation.
- The availability and marketing of unhealthy foods encourage their consumption among wealthier groups.
- Women from poorer backgrounds emphasised mainly government‐led interventions (e.g., job creation, affordable healthy food, childcare and improved cooking infrastructure), whereas those from wealthier backgrounds highlighted both government‐ and society‐led measures (e.g., urban agriculture and restrictions on unhealthy food availability and advertising). Gender equality in caregiving was noted in both groups.

## 1 Introduction

The double burden of malnutrition has emerged as a significant public health challenge in Sub‐Saharan Africa (Popkin et al. 2020). Recent analyses reveal a concerning rise in adult mortality linked to dietary risks and overweight/obesity, alongside persistently high (but decreasing) mortality from maternal and child undernutrition (Melaku et al. 2019). Despite remarkable progress, Ethiopia still has one of the highest rates of undernutrition amongst children under 5 years (U5) and women of reproductive age (WRA). Nationally, 40% and 5% of children U5 are stunted and wasted, respectively (Ethiopian Statistical Service ESS and ICF 2025) and 20% of WRA are anaemic, with 20% also underweight (Ethiopian Public Health Institute EPHI 2025). At the same time, overweight and obesity are rising, particularly amongst urban WRA (e.g., 36% in Addis Ababa), contributing to the double burden of malnutrition (Ethiopian Public Health Institute EPHI 2025).

Both forms of malnutrition have traditionally been considered distinct, requiring different interventions (Wells et al. 2020). The need to reshape public health nutrition interventions to address multiple forms of malnutrition simultaneously through double‐duty actions has been proposed (Hawkes et al. 2020a), but doing so requires a clear understanding of the problem and its drivers. Improving diets has been recognised as a key double‐duty action to address the double burden of malnutrition (Pradeilles et al. 2019; WHO 2017), and the need to understand the drivers of dietary behaviours to inform effective and context‐specific solutions in Ethiopia has been recently highlighted (Baye and Yaregal 2023).

In Ethiopia, diets are insufficiently diverse and the consumption of animal‐source foods and fruit and vegetables is low, particularly amongst WRA and children U5 (Ethiopian Public Health Institute EPHI 2025; Semagn and Abubakari 2023; Gebretsadik et al. 2022). Another study also indicated that the majority of WRA have a high to moderate risk of nutritional inadequacy and non‐communicable diseases due to poor quality diets (Baye and Yaregal 2023). In addition, the consumption of unhealthy foods and beverages is increasingly becoming a concern among infants and young children (Tizazu et al. 2024; Jemere et al. 2023; Ethiopian Public Health Institute EPHI 2025), adolescents (Berhane et al. 2023) and adults (Baye and Yaregal 2023), particularly in Addis Ababa, the country's capital city (Ethiopian Public Health Institute EPHI 2025). Similarly, while the overall contribution of ultra‐processed foods to total energy intake is relatively low nationally (2.4%), it is approximately five times higher in Addis Ababa (12.0%) compared with the national average (Balcha et al. 2025). Failure to improve the diets of WRA and children U5 could impede efforts to achieve the Sustainable Development Goals of ending all forms of malnutrition, reducing premature mortality from diet‐related non‐communicable diseases, ending poverty and achieving gender equality and women's empowerment.

To address poor‐quality diets, it is essential to understand the full spectrum of people's experiences and realities and how these interact to shape dietary behaviours (Hawkes et al. 2024). Such insights are essential to identify, design, implement and evaluate diet‐related policies and interventions (Hawkes et al. 2024; Spires et al. 2023). Photovoice is a recognised participatory method for generating evidence on lived experiences in food environment research in both low‐ and middle‐income countries and high‐income countries (Turner et al. 2023). In low‐ and middle‐income countries, Photovoice studies with school‐aged children, adolescents and women have consistently identified food affordability, accessibility, availability, convenience, and perceived quality and safety as key drivers of dietary behaviours (Wanjohi et al. 2025; Iyassu et al. 2024; Liguori et al. 2022; O'Halloran et al. 2021; Spires et al. 2021; Trübswasser et al. 2021; Pradeilles et al.^2021^). Social food environment and individual‐level factors such as peer pressure, social aspirations, income, nutrition knowledge, time constraints, cooking skills and reproductive status further shape dietary behaviours (Wanjohi et al. 2025; Posey et al. 2024; Wanjohi et al. 2023; Pradeilles et al. 2021). However, most of these studies focused primarily on identifying challenges to eating healthily, with limited attention to co‐creating solutions with participants. In Ethiopia, the use of Photovoice has so far been limited to understanding the drivers of dietary behaviours among adolescents (Iyassu et al. 2024; Trübswasser et al. 2021).

This study explored the lived experiences of WRA and their children U5 in urban Ethiopia, including women from both lower‐ and higher‐ socio‐economic status (SES) groups, to identify the challenges they face and the solutions they propose for consuming healthy diets for themselves and their children.

## 2 Methods

### 2.1 Study Setting and Design

This study is part of a wider project entitled TAMMIE (Tackling Multiple forms of Malnutrition In Ethiopia amongst WRA age and children U5) that aims to assess the causes of the double burden of malnutrition problem, and develop a roadmap for action to tackle it by characterising current policies and programmes, people involved and intervention priorities (CORDIS EU 2026). For this, a 4P cycle (*Problem, Policies/Programmes, People* and *Priority*) was applied, with a range of methodologies used to examine each component (Supporting Information S1: Material 1). This study fits under the *Problem* domain, which aims to understand the drivers of the double burden of malnutrition, specifically focusing on poor quality diets. It was conducted in Addis Ababa, the Ethiopian capital, which is home to nearly 5.7 million people, accounting for about 25% of the Ethiopian urban population (Ethiopian Statistics Services 2024).

To achieve the study's objective, we used a qualitative participatory photography method (Photovoice), which allows participants to use photographs to frame their problems and empowers them to describe their experiences and perspectives about public health problems (Wang 1999). The approach can engage individuals across diverse socio‐demographic backgrounds, including women and socio‐economically disadvantaged groups (Auma et al. 2024). Reflection and dialogue based on the photographs are used to identify community‐level priorities and ultimately create a platform for advocacy and social change (Duea et al. 2022). Therefore, this study invited women to showcase their photographs in focus group discussions (FGDs), explain their meaning, discuss perceived constraints to achieving a healthy diet, and propose potential solutions to address them.

### 2.2 Sampling

**Figure 1:**
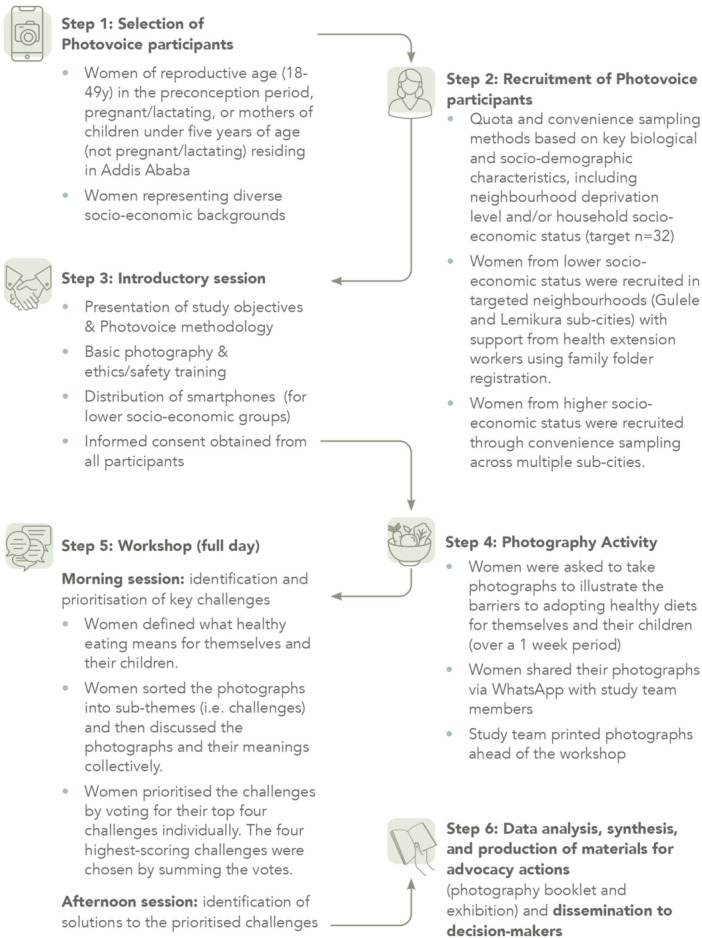
Steps of the Photovoice study.

WRA (18–49 y) were purposively recruited using quota or convenience sampling (Figure 1, steps 1 and 2), with a target sample of 32 participants across four FGDs. Target numbers were set according to life stage (i.e., pre‐conception, pregnant and lactating women and mothers of children U5) and socio‐demographic characteristics, including neighbourhood deprivation level and/or household SES, to ensure diversity of perspectives (Supporting Information S1: material 2). The target sample size also reflected pragmatic considerations, including available resources and logistical challenges in convening group discussions in urban settings.

To recruit women from lower SES, we targeted slum areas in Gulele sub‐city (Shiromeda and Gutomeda health centres) and Lemi Kura sub‐city (Woreda 02 health centre), selected based on their level of deprivation (Gelet et al. 2023). The selection of the study sites within the targeted neighbourhoods was informed by consulting with the sub‐city government office. Health extension workers at each health centre facilitated participant recruitment using family folder registrations, which included household SES classification and information on the woman's life stage and the age of children in the household.

To recruit women from higher SES, we ultimately relied on convenience sampling after unsuccessful attempts to reach them through health extension workers. Participants were recruited from different sub‐cities through the professional and community network of data collectors and included women who were willing to participate and met the life stage criteria. Higher SES was defined by educational attainment, home ownership and car ownership.

### 2.3 Data Collection

Data collection was conducted between July and November 2024. An introductory session (Figure 1, step 3) was held separately for each group to inform them about the study's objectives and train them in basic photography, ethics, and safety (Supporting Information S1: Material 3). Participants were asked to take 2‐4 photographs during 1 week to illustrate the barriers to adopting healthy dietary practices for themselves and their children (Figure 1, step 4). The training did not include defining ‘healthy diet’ to avoid influencing participants' knowledge and understanding. Smartphones were given to all women from lower SES, based on contextual knowledge from local partners, indicating limited access to devices or the internet. This facilitated participation, and women kept the phones at the end of the study. Women from higher SES used their own smartphones to capture photographs. Participants shared their photographs via WhatsApp with the study team members, who printed them ahead of the FGD workshop.

The FGD workshop (Figure 1, step 5) took one full day (see interview guide in Supporting Information S1: Material 4). In the morning, women were asked to define what healthy eating meant to them and their children. Then, participants sorted their photographs into sub‐themes (i.e., challenges) and discussed the photographs and their meaning collectively. The group discussions were guided using the PHOTO technique (could you talk about or describe your Photo; what is Happening in your photograph; why did you take a photograph Of this; what does this photograph Tell us; and how can this photograph provide Opportunities for positive change) (Wang and Burris 1997). Women then prioritised the challenges they faced, each selecting their top four challenges and providing justification for their choices. Within each FGD, the four highest‐scoring challenges were chosen by summing the votes across participants. Women then selected up to 10–15 of their photographs that related to the top four identified challenges and were asked to explain their choices. In the afternoon, women focused on proposing solutions to address these challenges. A four‐point matrix was used to guide the discussion on solutions, in which the participants indicated the idea (the proposed solution), who should implement it? (stakeholders responsible for implementing the solution), how should it be implemented? (proposed steps/processes of implementing the solution), and what is needed to implement the solution? (resources for implementation) (Savy et al. 2025).

We initially targeted 32 participants across four FGDs (*n* = 8 women per FGD), but some sessions included fewer participants, so an additional FGD was added to meet the target. A total of 31 of the 32 targeted women participated across five FGDs: three sessions with women from lower SES groups and two sessions with women from higher SES groups. Two additional participants attended the introductory session and submitted photographs, but were unable to attend the FGD workshop due to illness. Data collection was considered sufficient (Malterud et al. 2016) when all planned participant groups had been included, and FGDs were completed according to the study design, ensuring that data were adequate and sufficiently rich to address the study objectives, given the diversity of perspectives captured across SES groups and life stages.

All FGDs were facilitated by two women and one man, all native Ethiopians with Master's degrees and prior experience in qualitative research, including facilitating FGDs and Photovoice projects. Additional training and piloting of the interview guide were conducted before data collection to familiarise the team with the study objectives and interview guide and to identify any necessary amendments. FGDs were conducted in Amharic, separately for women in the relatively lower and higher SES groups. All group discussions were digitally recorded and transcribed/translated (from Amharic to English) verbatim by one member of the team of data collectors fluent in both languages. Quality checks were performed on the translations and transcriptions by MW and RP.

### 2.4 Data Analysis and Synthesis

A hybrid thematic analysis (Figure 1, step 6) was conducted, with initial deductive coding informed by predefined frameworks, followed by inductive identification of emerging themes and sub‐themes (Braun and Clarke 2006). For the deductive thematic analysis, we used existing global food system and African food environment frameworks (Food systems dashboard FSD 2025; Osei‐Kwasi et al. 2021) to organise and categorise the themes and sub‐themes. The Food Systems Framework comprises four key components: *individual* factors that influence food choices (e.g. economic, cognitive, aspirational, situational and behavioural); *food supply chains* (e.g. from food production to distribution to processing and markets); *food environments* (e.g. food availability, affordability, product and vendor properties and food messaging), and the external *macro‐level drivers* that affect these processes (e.g. politics and leadership, socio‐cultural dynamics, migration and conflict), outcomes (food security and diets), and impacts (on the environment, nutrition and health outcomes, social equity and the economy) (Food systems dashboard FSD 2025). The codebook was structured around the four components described above. To address gaps in the original framework, such as limited emphasis on the social food environment, we also drew on the African Urban Food Environment Framework to further categorise themes and sub‐themes under each of the four components (Osei‐Kwasi et al. 2021). For example, under *food environment*, we organised the data into social and physical food environments, with the latter being further split into home, neighbourhood, school and workplace food environments. Influences within the social food environment (e.g., family, peers and local food vendors) were captured under these neighbourhood‐level components, while macro‐level socio‐cultural drivers, such as norms and traditions shaping dietary behaviours, were captured under macro‐level drivers. An inductive thematic analysis was then conducted to identify additional themes and sub‐themes emerging from the data. The codebook was piloted, reviewed and amended several times before its use. Once the codebook was finalised, the data were coded using NVivo 14 by MW (Ethiopian PhD candidate in Community Nutrition) and AI (Researcher in Global Health Nutrition with extensive qualitative research experience). Quality checks were performed by RP (Senior Researcher in Global Health Nutrition with extensive qualitative research experience and Ethiopian heritage) on all transcripts.

The synthesis focused on the prioritised challenges and their proposed solutions, with findings presented separately for the lower and higher SES groups. Each FGD initially prioritised four challenges per session, which were then combined within each SES group. Repeated challenges were counted once, while unique challenges were added to the final priority list for that group. Low income and unemployment (individual level), financial inaccessibility (food environment level) and high cost of living (macro‐level drivers) cut across several levels of the framework, but all relate to the same overarching issue and are interconnected; they were therefore merged and presented under economic drivers. Social‐cultural factors at the macro level, as well as social food environment influences (limited in this study to family or spouse influences) were considered alongside individual‐level factors, particularly in shaping unhealthy food preferences and behaviours, including those related to time constraints. No additional macro‐level drivers were discussed beyond socio‐cultural influences and the high cost of living.

The final step (Figure 1, step 6) in the Photovoice study also involved the preparation and dissemination of advocacy materials, including a photography booklet (Pradeilles et al. 2025) and a photography exhibition held during the final stakeholder dissemination workshop. Results were presented to key decision‐makers from government, UN agencies, nongovernmental organisations (NGOs), academia and research institutions, and the private sector (*n* = 20) who are actively working in Ethiopia. Stakeholders were asked to vote for the three photographs that resonated with them the most and to share their reflections on the challenges requiring urgent attention, as well as potential solutions.

### 2.5 Ethics Statement

Ethical approval for the study was acquired from the Ethiopian Public Health Association (EPHA) Ethics Review Committee (EPHA/OG/331/24). The Ethics Review Committee granted permission for reuse of photographs in scientific outputs. Written informed consent was obtained from all participants. A photograph release form was used to request consent to take photographs if a person's face was visible and participants consented to photographs being used in scientific outputs.

## 3 Results

### 3.1 Socio‐Demographic Characteristics of Participants

Overall, women from all targeted life stages were equally represented. The mean age was 30 ± 6 years, with half between 25 and 34 years. Two‐thirds of women were married (64.5%), three‐quarters were working (77.4%), and over half had been educated above secondary school (58.1%) (Table 1).

**Table 1:** Socio‐demographic characteristics of study participants.

	Total		Lower SES*		Higher SES**	
	Frequency (n = 31)	%	Frequency (n = 20)	%	Frequency (*n *= 11)	%
Age (years)						
15–24	6	19.4	4	20.0	2	18.2
25–34	16	51.6	12	60.0	4	36.4
35–44	9	29.0	4	20.0	5	45.5
Life stages of women						
Pre‐conception	8	25.8	5	25.0	3	27.3
Pregnant	7	22.6	5	25.0	2	18.2
Lactating with a child under 2 y	8	25.8	5	25.0	3	27.3
Lactating or not with a child 2–5 y	8	25.8	5	25.0	3	27.3
Educational level						
No education	1	3.2	1	5.0	0	0.0
Primary education	3	9.7	3	15.0	0	0.0
Secondary education	9	29.0	9	45.0	0	0.0
Above secondary education	18	58.1	7	35.0	11	100.0
Occupational status						
Not working	7	22.6	7	35.0	0	0.0
Unskilled manual	7	22.6	7	35.0	0	0.0
Skilled manual	4	12.9	4	20.0	0	0.0
Professional/technical	13	41.9	2	10.0	11	100.0
Marital status						
Not married	7	22.6	4	20.0	3	27.3
Married	20	64.5	12	60.0	8	72.7
Divorced/separated	4	12.9	4	20.0	0	0.0

* Lower SES (socio‐economic status): Women classified as deprived/poor and nutritionally vulnerable on the family registration book.

** Higher SES: Women with higher educational attainment, who own a personal car and a house.

### 3.2 Perceived Challenges of Adopting Healthy Diets Among Women and Proposed Solutions

Key priority challenges were identified across the workshops, with six challenges prioritised in the lower‐SES groups and five in the higher‐SES groups, selected from a total of 11 and 17 challenges, respectively (Supporting Information S1: Material 5). To represent these priority challenges, women in the lower SES groups selected 26 photographs from an initial pool of 87, while women in the higher SES groups selected 16 photographs from an initial pool of 39.

**Figure 2:**
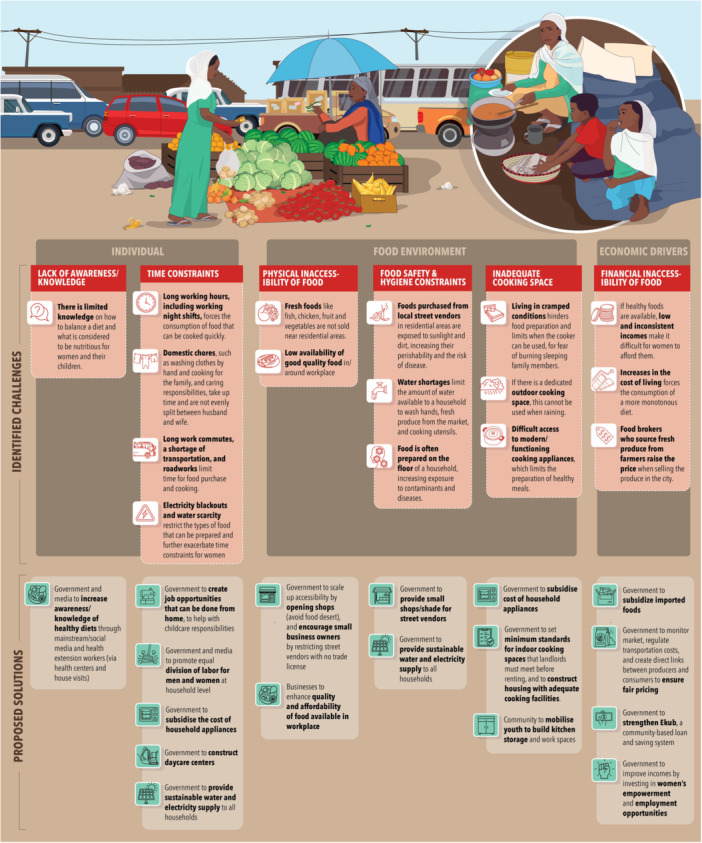
Perceived challenges and proposed solutions to adopting healthy diets amongst women of reproductive age and children U5 in lower‐income groups.

**Figure 3:**
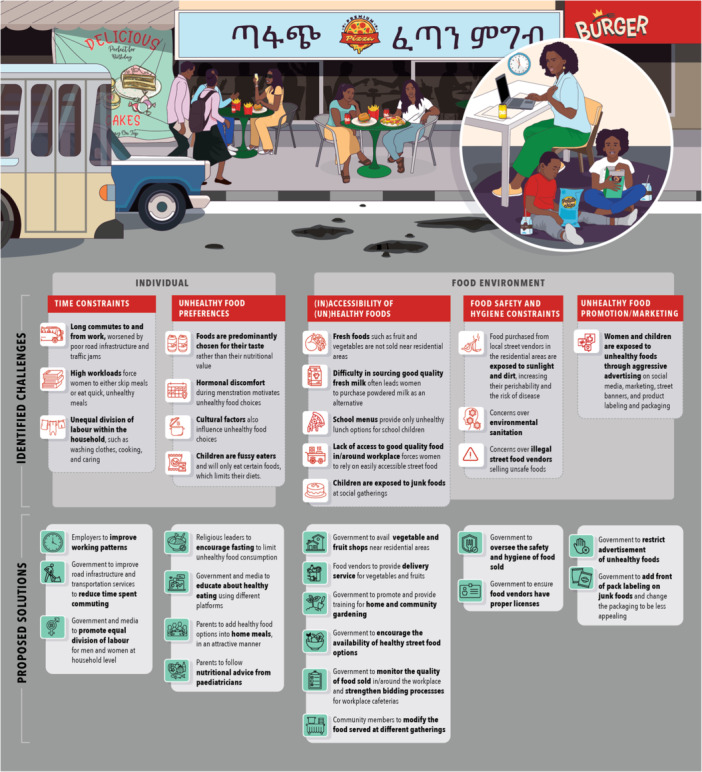
Perceived challenges and proposed solutions to adopting healthy diets amongst women of reproductive age and children under five in higher‐income groups.

Challenges identified by study participants included individual‐level barriers (e.g., lack of knowledge about healthy diets, time constraints, low income, and unhealthy food preferences); neighbourhood food environment constraints (e.g., physical inaccessibility of healthy foods, poor food safety and hygiene, and unhealthy food promotion); home food environment barriers (e.g., inadequate cooking space and kitchen appliances); and economic constraints (e.g., inflation, high food prices, under‐or unemployment, and the rising cost of living). Women suggested a mix of government, community, and market‐based interventions to address the prioritised challenges to adopting healthy diets for women and children. These recommendations, alongside the challenges, are summarised and illustrated in Figures 2 and 3.

#### 3.2.1 Individual‐Level Factors

**Figure 4:**
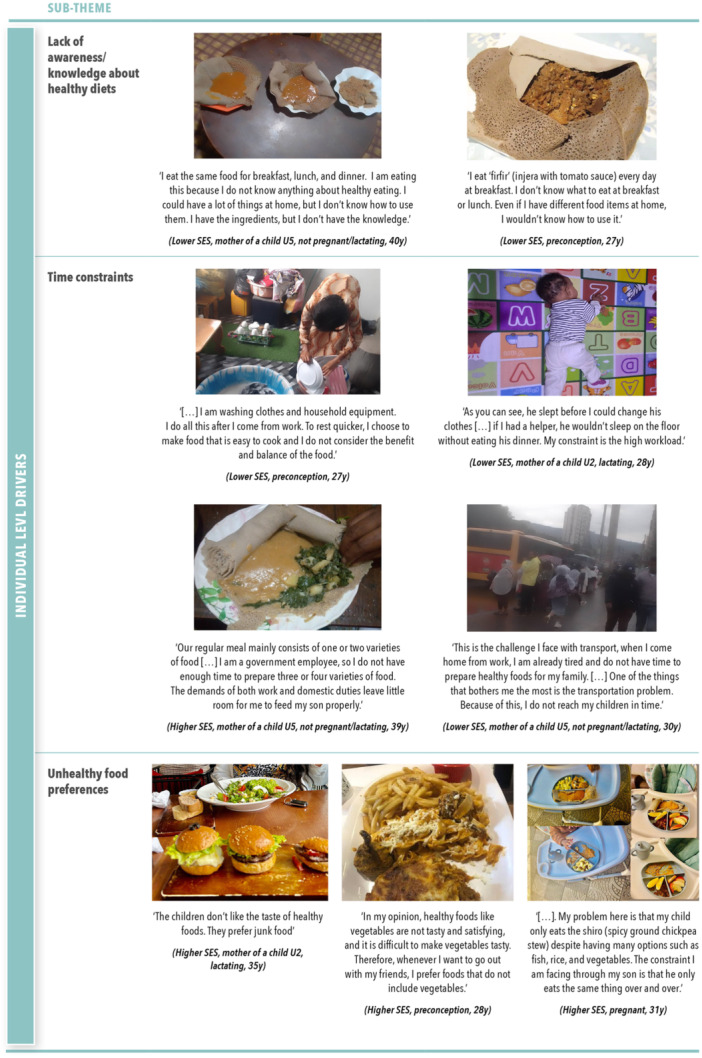
Individual‐level challenges to adopting healthy diets across socio‐economic groups.

- Lack of awareness/knowledge about healthy diets
  Participants from lower SES reported that while they have food at home and they are aware of what constitutes a nutritious diet, their lack of knowledge about combining different foods and creating balanced meals limits their ability to provide healthy meals for themselves and their children. As a result, they often eat traditional monotonous diets (Figure 4).
  A solution raised by women was to increase the level of knowledge and awareness about healthy diets using different platforms, including national television and social media. Health extension workers in the *woredas* (district) were also regarded as key entry points to deliver nutrition education programmes through community gatherings. In addition, women suggested integrating nutrition education with existing health education programmes at health centres.
  It will be good if messages on healthy eating are aired on television or social media. […].
  (Lower SES, mother of a child U5, not pregnant/lactating, 36 y)
  The health centre teaches mothers who come in for treatment. The treatment will start after the lesson is over. But in this case, it would be good to include the issue of healthy eating in those lessons.
  (Lower SES, mother of child U5, not pregnant/lactating, 36 y)
- Time constraints
  Women from both lower and higher SES groups identified time constraints as a major barrier to healthy eating, largely due to unequal division of labour within the household, lack of support, multitasking, long working hours, and the nature of their jobs (Figure 4).
  Most women from lower SES stated that they bear the full burden of household responsibilities, while often working long hours. Without proper support from their husband (or other family members) participants noted that they struggle to balance work, child caregiving, and cooking. As a result, they often prioritise meals that are quick to prepare and monotonous over meals with higher nutritional value. Women who struggled to find time to feed their children reported skipping their own meals, exacerbating feelings of being overwhelmed. Lack of convenient and fast transportation was also mentioned as a challenge for women of lower SES, affecting their ability to care for family and use their time efficiently.
  Similarly, many women from higher SES groups struggled to maintain a healthy diet due to busy work schedules, studying, or running a business. Government‐employed women are challenged with finding time for cooking and childcare due to demanding jobs. Some skip meals or rely on fast and convenient foods available at nearby vendors over healthier alternatives. For most higher‐income women, cooking at home is challenging due to a lack of time and energy, limiting the variety of home meals consumed.
  Women from both lower and higher SES groups proposed several solutions to address time constraints. These included recommending that the media promote equal division of labour, including childcare, between men and women within households, and that the government enhance working conditions, such as reducing working hours or adjusting working patterns (e.g., by adjusting lunch breaks or implementing shift schedules). On gender equity, women from lower SES strongly believed that upbringing is key in shaping husbands' attitudes and perspectives on sharing household chores. Hence, women proposed gradual cultural changes within their family and wider community to create a more gender balanced and supportive environment.
  From now on, families must take responsibility. When raising our children, we should teach them equal responsibilities, avoiding gender‐based task divisions. We shouldn't say, ‘This is for boys’ and ‘This is for girls’.
  (Lower SES, mother of child U5, not pregnant/lactating, 40 y)
  There needs to be a division of labour. When I have too much load, he has to be able to cover for me. I have to give time for my family, so we could try help each other.
  (Higher SES, mother of a child U5, not pregnant/lactating, 37 y)
  Having better support systems, including assistance (e.g., household helper) and childcare and nutrition services such as day care, was seen by women from lower‐ and higher‐SES groups as a key solution. They suggested that the government should improve the accessibility and affordability of day care services; while employers should consider offering better work schedules, which would allow women to have more time to care for children.
  I have two children, so I can't go out to work, and even if I could, the question of whether my income would be enough to cover childcare service expenses is a question. Childcare services should be more available around where we live.
  (Lower SES, mother of a child U2, lactating, 28 y)
  I am working two shifts. I have some time in the middle of the shifts, but when I try to get back home, most of the time is spent on the road. But if I were on one of the three shifts, there would be enough time to feed my children and to cook also.
  (Lower SES, mother of a child U2, lactating, 31 y)
  Women from higher SES groups proposed reducing the time spent commuting (e.g., by improving roads and infrastructure, traffic congestion, and transportation services).
- Unhealthy food preferences

Women from higher SES groups reported that food preferences were a key driver of unhealthy diets for them and their children. Mothers reported that children normally refuse to eat a diverse diet as children prefer so called ‘junk foods’ over healthier food options. To encourage better eating habits, women proposed presenting food in an enjoyable and engaging way, introducing healthy foods at the right time (e.g., by offering new flavours early on), or allowing children to be actively involved in their own feeding (Figure 4).

Children first eat with their eyes. So, if we present the meal with good, attractive plating, and maybe let them ditch the spoons and eat with their hands, it will be good. We can also cut the food into shapes to persuade them to eat healthy.

(Higher SES, mother of a child U2, lactating, 35 y)

Although most women from this group generally displayed good knowledge of what a healthy diet constitutes and its importance during different life stages (e.g., pregnancy and lactation), some also expressed having unhealthy dietary behaviours due to cultural beliefs, e.g., consumption of locally made alcoholic drinks to increase breast milk volume. Similarly, menstruation‐related symptoms, including cravings for unhealthy foods (e.g., fast foods and sweets) were reported to disrupt their eating habits and influence their mood. Other women mentioned prioritising personal food preferences (e.g., consuming high‐fat foods that are more satisfying) over healthier, less appealing foods like vegetables who are less tasty. Women admitted that changing their own attitude towards healthy eating was key to improving their dietary behaviours and that of their families.

The first thing is convincing ourselves. If you try to work on behavioural change of someone, it is a problem if that person is not ready. It is not just a lack of knowledge, but even though they know, it is also not giving it attention and not practicing it.

(Higher SES, pregnant, 31 y)

At the community level, possible initiatives proposed by women of higher SES was to raise the level of knowledge and improve dietary practices. These included engaging community and religious leaders to help shape eating habits through fasting practices and education; involving health professionals (e.g., nutritionists) to educate people on healthy eating based on scientific research to address false beliefs; and integrating of nutrition education into the school curricula, starting early and involving parents. Further, due to its influence on dietary choices, women proposed that the Ministry of Health should work collaboratively with health professionals to oversee the content of nutrition‐related videos shared online and on TV/radio to ensure their accuracy and accessibility for urban and rural populations (e.g., using simple language).

We are better at accepting things that come from higher‐up. It could be from the government or a religious organisation.

(Higher SES, mother of a child U5, not pregnant/lactating, 37 y)

These days, we get our information from TikTok and social media. The content we consider true about nutrition for mothers or children should be overseen by the Ministry of Health. That content reaches people in cities, but those who live in rural areas of the country go to healthcare service providers around them. So, making some easily understandable content for them and for those who can't read and write, teaching them, and giving them flyers is a good idea.

(Higher SES, pregnant, 31 y)

#### 3.2.2 Food Environment Factors

**Figure 5:**
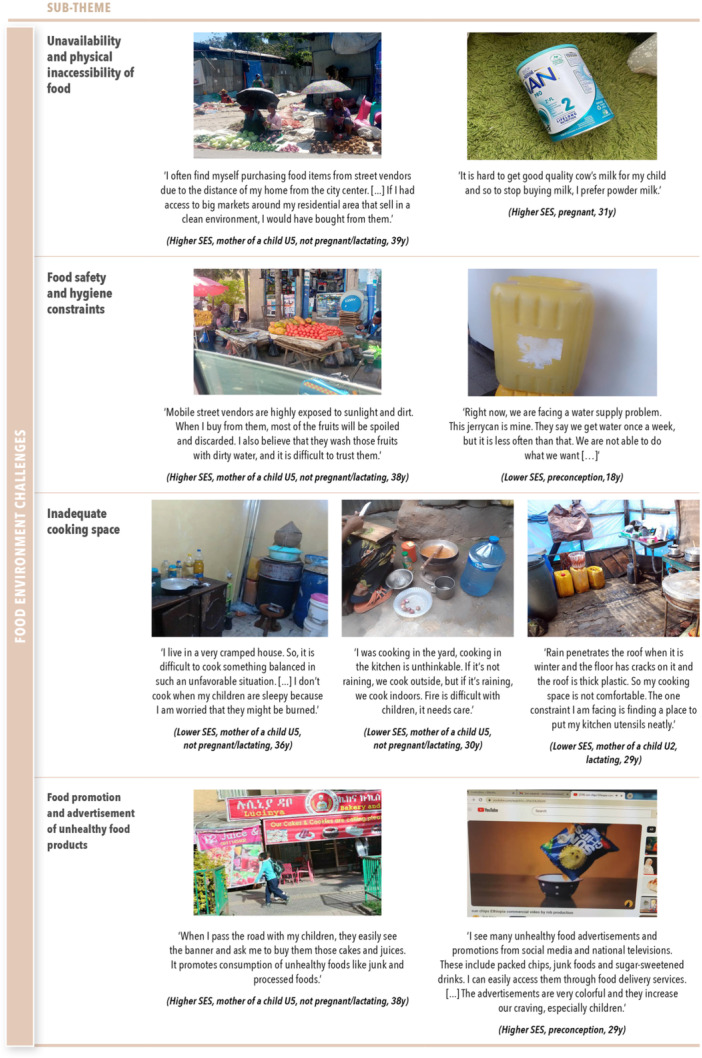
Food environment challenges to adopting healthy diets across socio‐economic groups.

- Challenges with the availability and physical accessibility of (un)healthy foods
  In relation to the *neighbourhood food environment* (Figure 5), some women from lower SES identified the limited availability of certain desired foods (e.g. fresh foods like chicken, fruit and vegetables) as a key constraint to achieving dietary diversity and quality. Nonetheless, the majority of participants reported that basic food items (e.g. potatoes, onions, carrots, and tomatoes) were generally available at their local food markets. Women from the higher SES group noted the presence of nearby food outlets and delivery options that facilitated food purchasing. However, living far from the city centre restricted their access to large‐scale formal markets and supermarkets. As a result, women often relied on smaller local markets and street food vendors, where fresh produce was often less available and varied in quality and hygiene. While women appreciated nearby marketplaces that operate on weekends and sell fruit and vegetables at lower prices than regular shops, purchasing from small markets was also reported to be inconvenient, due to the need to visit multiple vendors. Mothers also noted that the widespread availability of junk food, sugary drinks, and processed snacks in nearby shops and food delivery services posed a significant barrier to healthy eating. In addition, limited access to good‐quality fresh milk for their children was noted as a challenge, which led mothers to purchase powdered milk, despite its high cost. 
  Women from both SES groups suggested that the government improve access to large food markets in local areas (*woredas*) and to other types of shops. Women from higher SES additionally emphasised the need to improve the quality of food sold in their neighbourhood. They also suggested the establishment of a delivery service for vegetable vendors to enhance access to fresh produce, with the government and private sector identified as key implementers. According to them, the government should also promote local food production instead of food imports and support urban agriculture with enhanced technology to make healthy food more accessible and affordable.
  We can do poultry at our house as well. We could then easily get eggs and white meat, which is healthy. The government should support urban agriculture using different technologies. If we need to buy chicken from supermarket, it is very expensive for most people, but through own production, we can afford it.
  (Higher SES, preconception, 28 y)
  Home gardening (i.e., growing vegetables at home and use of vertical farming if space is limited) and community gardens were also mentioned by women from higher SES groups as a sustainable solution to ensure easy access to fresh vegetables.
  We have to find out our own solution as well. We can start own production. We can grow vegetables around our house. If we do not have an adequate place, we can use vertical farming.
  (Higher SES, pregnant, 31 y)
  Within the *neighbourhood food environment*, social events and gatherings were also reported to hinder healthy eating due to the excessive presence of unhealthy foods, e.g. sugary foods/drinks. Women suggested that healthier food options should be available at such occasions, for example, offering fruit, oat‐based pancakes and barley flour cakes to make celebrations both nutritious and visually appealing.
  Changing the catering options with fruit, using oats for pancakes, so when we order, it will also be good as a variety, good for a picture, and good for health. We could also request the cake to be baked using barley flour.
  (Higher SES, preconception, 22 y)
  With regards to the *work food environment*, women of lower SES had a wide range of views on food availability and quality at work, which varied depending on job type and distance from home. For example, women who were working in professional or technical jobs reported having access to different spaces and services within their workplace to eat, such as cafeterias and special discounts. Women experiencing challenges in accessing good‐quality food at their workplace, and those working near their homes, opted to prepare meals (or eat) at home, when possible, to ensure better nutrition. However, participants working far away from home often purchased food near their workplaces, which tended to be cheap but low‐quality. Similarly, women of higher SES reported having limited healthy options and plentiful access to unhealthy foods through food vendors near work. Those with access to a work cafeteria complained about the poor quality and sanitation of the food sold. In both contexts, women explained the bidding process for food providers who often prioritise price over quality. Hence, women proposed changes that include monitoring the quality of food available at their workplace, as well as to consider the quality and healthiness of the food in the contracting process.
  The cafeteria is temporarily owned by the bid winner. When we evaluate the bid participants, we only see the price, but we have to consider the quality as well. When we approve the winner, we should consider the quality and healthiness of the food.
  (Higher SES, mother of an U5 child, not pregnant/lactating, 39 y)
  Lastly, regarding the *school food environment*, women from higher SES groups mentioned that schools dictate what children can bring for lunch and snacks and currently promote unhealthy food consumption. Women reported that there was some flexibility for children with certain conditions and that the programme generally ensured uniformity to reduce disparities. However, they felt frustrated by the lack of healthier food options in schools.
- Food safety and hygiene constraints
  Within the *neighbourhood food environment*, concerns about food safety and hygiene when purchasing and supplying food products from different outlets emerged in both SES groups. Women of lower SES raised concerns about food adulteration and hygiene practices affecting the quality of purchased foods/meals (e.g., poor handling, fluctuating temperatures and unsanitary conditions used during transportation), describing homemade food as safer. For example, women mentioned that *injera* (fermented flat bread made from *teff* flour, the staple cereal grain in Ethiopia) sold in their neighbourhoods sometimes contains added rice and wheat to reduce costs. Women of higher SES also raised issues regarding food safety and hygiene, especially in the context of environmental sanitation, i.e., the cleanliness of spaces where food is prepared and cooked (Figure 5).
  Overall, foods sold at supermarkets and well‐established markets were considered to be more hygienic and, hence, of higher quality when compared to local street markets, which expose foods to adverse weather conditions, increasing perishability. Nevertheless, financial constraints force many women, particularly those from lower SES, to purchase food from street markets.
  One solution proposed by women of lower SES was for the government to construct shelters for street vendors to enhance the cleanliness and quality of food products; whereas women of higher SES suggested that the government should monitor the safety and hygiene of food sold, noting that many street vendors operate without proper licenses.
  If the government makes them small shades, the dust and cleanliness of the items could improve. That could result in them selling a better‐quality product.
  (Lower SES, pregnant, 28 y)
  The government should properly monitor the health of street foods. Most street vendors are illegal, and they do not have a license to work.
  (Higher SES, preconception, 28 y)
  Within the *home food environment*, women of lower SES underscored the importance of food hygiene (i.e., the “cleanliness of food”) and environmental sanitation for a healthy diet and well‐being. Some women stated that food is prepared on the floor, which increases exposure to contaminants, which was highlighted as a particular risk during pregnancy. Compounding this, water scarcity was raised as a challenge by women, impacting their personal hygiene, food safety, ability to cook and clean, with some women avoiding foods that require washing. To cope with this, one participant mentioned storing water from neighbours, as she does not have consistent access in her household.
- Inadequate cooking space, household utilities and facilities
  Women of lower SES tend to live in small and cramped spaces, making cooking difficult and unsafe, especially for mothers with young children. Many women therefore opt to cook outside due to inadequate infrastructure, e.g., leaking roofs, unstable flooring, or poor ventilation. These conditions are particularly challenging during bad weather or at night (Figure 5). Similarly, women reported that the lack of modern cooking appliances (e.g., ovens, stoves, or juicers) further hinders the cooking process and limits healthy meal preparation. These challenges are exacerbated by frequent electricity outages and water shortages. Power cuts force women to rely on alternative fuels, such as using coal, which they describe as ‘time‐consuming’, ‘physically exhausting’, and ‘hazardous’ due to smoke inhalation.
  These challenging cooking conditions led to significant emotional distress among women, manifesting in frustration, guilt, and stress. During discussions, women proposed that, instead of relying on financial assistance, the government should lower the price of cooking appliances and other household utilities (e.g., electric stoves and washing machines). Additionally, women mentioned the traditional ‘*ekub*’ system—a neighbourhood‐based saving practice that helps women acquire necessary items—and emphasised the need to strengthen and expand this support network.
  Mothers like us, we are raising children and we have no helpers. We can't afford to hire helpers, we are not asking for funding directly but rather discounting and supplying our living area with materials that would help.
  (Lower SES, mother of an U2 child, lactating, 28 y)
  Yes, in some neighbourhoods, there is a thing called an item ‘*Ekub*’. It is not the money, but the item that is bought and given to you.
  (Lower SES, mother of a child U5, not pregnant/lactating, 36 y)
- Unhealthy food promotion and advertisement of unhealthy foods

Women of higher SES brought up the topic of food advertising as a challenge to healthy eating. They mentioned different channels, including social media, TV, banners on the streets, or labelling and packaging of unhealthy products. Participants also mentioned that, as the promotion of healthy food products is rare, it is difficult to counterbalance the influence of unhealthy food advertising. Women believed that colourful and frequent advertisements influenced their children's food preferences, leading to the consumption of sweet, processed, and packaged foods (Figure 5).

For potential solutions, women suggested that the Ministry of Health could revise current policies and restrict or ban the advertising of unhealthy foods, e.g., by labelling these products with warnings similar to those on alcohol and cigarette packaging, allowing people to make informed choices when purchasing food. Likewise, women raised the importance of making packaging less appealing for children, especially for unhealthy foods. Participants referred to a recent successful initiative requiring infant formula packaging to include a nutritional label stating the superiority of breastmilk and recommending it as the first choice.

The Ministry of Health should play their role. We see there are messages on alcohol drink bottles because it is a must. So, when we come to the food, problematic meals like burgers and takeaway‐packed [‘junk’] foods should also be expressed as harmful for the body, the people could choose to eat or not to. Just like on cigarettes and alcohol beverages, these meals should also be labelled as well.

(Higher SES, mother of a child U5, not pregnant/lactating, 37 y)

Now, there's an action taken against infant formula, which prevents its advertisement and promotion. Packages of flavoured milk drinks are so appealing with their straws. I try making strawberry milk at home, but my child doesn't like it as much. She's attracted to the packaging and the colours she sees there. Even when I offer her both, she chooses the factory‐made artificial one over the natural one. The same measure has to be applied to flavoured milks as well.

(Higher SES, mother of a child U2, lactating, 35 y)

#### 3.2.3 Economic Drivers

**Figure 6:**
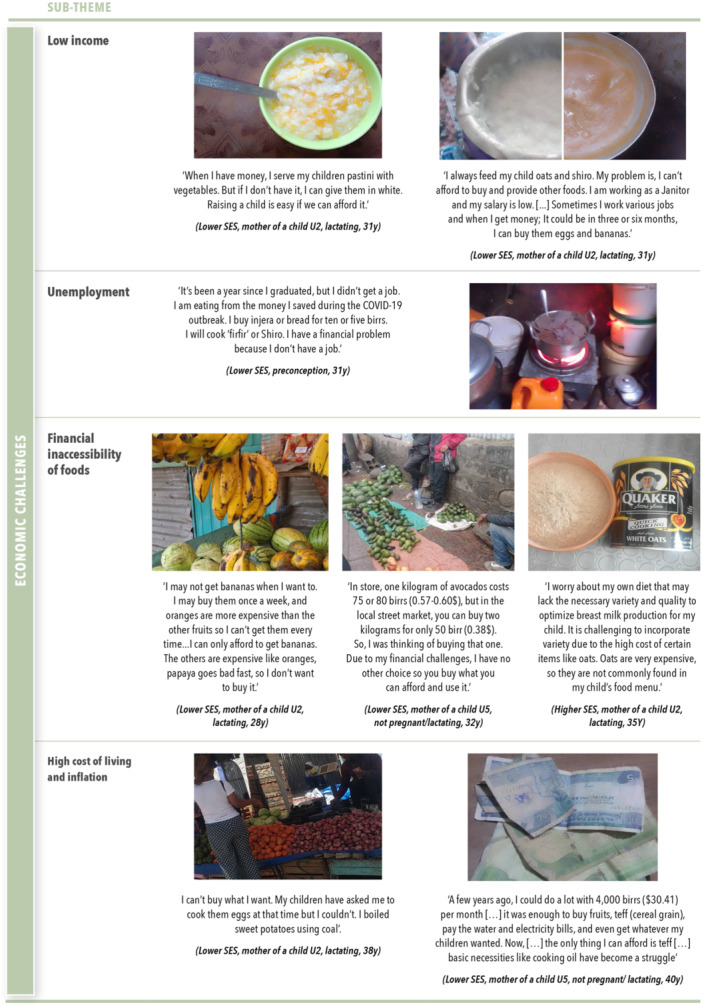
Economic challenges to adopting healthy diets across socio‐economic groups.

- Poverty and unemployment
  Women of lower SES expressed significant challenges in affording essential food items, household appliances, and basic services (e.g., water and electricity) for themselves and their families. These challenges were often linked to unemployment, limited job opportunities, low pay, and the rising cost of living (including the price of essential foods, such as meat, eggs and fruit). Working mothers noted that their purchasing power had significantly decreased over time, despite having the same income and now struggle to buy basic foods (Figure 6).
  To cope, some women reported working multiple informal jobs, such as selling vegetables on the roadside. Others expressed appreciation for the government initiatives providing subsidised bread and the food assistance programmes available through charities. Women recommended that the government also subsidise household appliances, which could help them save time and diversify their diet. Additionally, a few mothers mentioned their need to start small businesses to improve their financial situation but cited a lack of capital as the main constraint.
  Many women of lower SES discussed struggling to find a job, relying on their savings, and managing limited money for staple foods, like *injera*. Proposed solutions included government‐led initiatives to create job opportunities and improve access to essential resources and spaces to help women create and strengthen their businesses to achieve financial independence. Additionally, women suggested that home‐based or remote jobs would be a more effective solution, allowing them to earn an income while caring for their children and families. Lastly, salary increases, additional income opportunities, and flexible work arrangements (e.g., part‐time) were also raised by women to ensure a better standard of living.
  If I have the opportunity to work at home, I will be more effective, I can take care of my mother and child. In that way, my challenges can be solved. But I can't solve all these challenges by myself.
  (Lower SES, mother of a child U5, not pregnant/lactating, 30 y)
  I tried to open a juice bar so many times. There is rent, work permit too. It is all expensive. It would be good if a government body supported us.
  (Lower SES, mother of a child U5, not pregnant/lactating, 25 y)
- Financial inaccessibility of healthy foods
  Women from lower SES groups reported that financial hardship, coupled with the high cost of nutritious foods, prevented them from buying a variety of nutritious and essential foods. Meat, eggs, and certain fruits were often unaffordable. Despite their awareness and knowledge around healthy eating, many women reported relying on cheaper, less nutritious foods like bread, tea, *shiro (ground chickpea stew)*, and packaged juices. Some also mentioned frequently skipping meals due to finances (Figure 6).
  Women appreciated a government initiative intended to offer lower‐priced goods, but noted that prices were generally similar to those in regular markets. Additionally, women mentioned that the quality of foods from government‐funded shops was poor; hence, they suggested improving the quality of products and reducing their price. Constraints around food affordability were sometimes mitigated through special services offered by food vendors who allowed customers to pay monthly rather than daily, or through food coupons and affordable prices proposed at workplace cafeterias.
  I think the government has to fix this. Even though they sell vegetables and fruits in the market, the prices posted at the government shops are the same as in other shops. So, if they adjust the prices, we could buy good quality produce at an affordable price.
  (Lower SES, pregnant, 28 y)
  Whilst it was not prioritised in the top four challenges, women of higher SES also acknowledged that the cost of food had increased and that, as a result, adopting a balanced diet had become more challenging.
- High cost of living and inflation

Women from both SES groups noted the rising cost of living while income remained unchanged, further worsening financial challenges. Women of lower SES highlighted rising house rental, utilities and transportation costs leaving families with limited money for food and other necessities. They also mentioned the high cost of food, which further constrains family food budgets. They attributed the price difference between farmers and consumers to brokers and transportation costs, which significantly increase food prices in cities like Addis Ababa. Additionally, illegal imports and price manipulations by retailers contribute to inflated costs. As a result, women mentioned choosing cheaper foods, such as potatoes, onions, carrots, and tomatoes, instead of more expensive options such as broccoli and meat. Street vendors offer more affordable prices compared to shops and supermarkets, but financial constraints still limit the quantity and variety of purchases (Figure 6).

To alleviate the financial burden, women emphasised the need for government interventions to monitor and regulate the market by connecting producers and consumers to ensure fair pricing and regulating transportation costs.

If the government has a system that connects the producer and the communities directly and there is no broker in the middle, if we can buy directly from the farmer, it will reduce our living expenses.

(Lower SES, pregnant, 28 y)

The government should monitor the food chain process from farmers to consumers. I think it would be good if there were a fixed price for farmers to sell their products. Then, by adding a reasonable transportation fee, the merchants could sell at a lower price.

(Lower SES, mother of a child U5, not pregnant/lactating, 36 y)

### 3.3 Stakeholders' Views on Priority Challenges and Solutions

In the final dissemination workshop, stakeholders emphasised the need for financial actions, including strengthening social safety net programmes targeting the urban poor to improve accessibility to and consumption of healthy foods. They also noted that taste is acquired early in life and that parents, particularly mothers, play a central role in shaping children's food preferences, highlighting the importance of nutrition education delivered through different platforms. Some stakeholders were particularly struck by women's emphasis on the need for adequate cooking space to prepare nutritious meals and ensure food safety. They suggested that community‐level interventions, such as establishing communal kitchens in economically deprived areas, could help improve food safety while reducing the time required for meal preparation.

## 4 Discussion

Using Photovoice, the current study provides insights into the perceived challenges and proposed solutions for achieving healthier diets from the perspective of urban Ethiopian WRA from lower‐ and higher‐SES groups. To address barriers to adopting healthy diets, women in our study proposed a diverse set of government‐ and societal‐level solutions. These solutions spanned multiple domains, including food environment, financial support, education, and gender equity. Government‐level solutions were primarily raised by women of lower SES, reflecting the limited presence of agencies working closely with the urban poor and reinforcing the perception that the government is the primary actor responsible for addressing their challenges.

### 4.1 Individual‐Level Challenges and Proposed Solutions

*At the individual level*, time constraints were consistently reported as a major barrier to consuming healthy diets across both SES groups. Women were responsible for multiple tasks, including meal planning, cooking, childcare and income‐generating work, with close to three‐quarters of women employed outside the home. The lack of support from spouses or other family members further compounded time pressures, often leading to the preparation of quick, convenient, or monotonous meals. For women in higher SES groups, employment and long commuting time further contributed to time constraints and were often cited as a reason for purchasing unhealthy and convenient food near their workplaces. Similarly, in India, the lack of family support, combined with the need for women to manage multiple responsibilities, was identified as a key factor driving women's purchases of ready‐to‐eat food items (Chaturvedi et al. 2016), and convenience and quick food preparation were important drivers for choosing ready‐to‐eat foods (Venkateshmurthy et al. 2021).

Women from lower SES backgrounds reported additional constraints, including limited knowledge about healthy diets, which further affected their ability to adopt healthy diets. A mixed‐method study conducted in Ethiopia identified limited maternal understanding of healthy eating as a contributing factor to unhealthy diets among adolescents (10–14 y) (Berhane et al. 2023), which aligns with our findings.

Women of higher SES also reported that unhealthy food preferences, partly shaped by exposure to unhealthy food advertising, were important drivers of their dietary behaviours. Taste strongly influenced their satisfaction and overall intake, consistent with findings from other studies highlighting the significant role of taste in food selection (Liem and Russell 2019) and consumption of unhealthy food (Iyassu et al. 2024; Liem and Russell 2019). Mothers also emphasised that taste shaped their children's food preferences. Similarly, a study in Ethiopia found that mothers tend to give children their preferred food, out of concern that the child may not eat otherwise (Berhane et al. 2023). Evidence from Ghana and Kenya further suggests that women play a central role in influencing household members' dietary behaviours, by considering both the nutritional needs and preferences of their family members when purchasing foods and preparing meals (Wanjohi et al. 2023).

To address individual‐level drivers, women proposed key solutions. Strengthening n*utrition education* programmes was proposed as a key solution to counter the influences of unhealthy food advertisements and to increase knowledge and awareness about healthy diets for women and children. This could be achieved by delivering culturally‐appropriate nutrition education through mass media and/or social media, behaviour change communication campaigns, and dietary counselling to women during the childbearing period (Hawkes et al. 2020b), aligned with the Ethiopian food‐based dietary guidelines (Federal Government of Ethiopia, Ethiopian Public Health Institute, & Ministry of Health 2022). In addition, women emphasised the importance of *promoting gender equality*, particularly in caregiving responsibilities. Ethiopia has made progress on gender equality in recent years, through the introduction of various policies, though gaps remain in economic, educational, and household domains (MOWCY, UNICEF Ethiopia and SPRI 2019). The additional constraints reported by women from lower SES backgrounds, including limited income, unemployment and inadequate access to resources, reflect these broader structural and labour market inequities. Addressing such disparities by reducing gender inequities in household and caregiving responsibilities, and improving women's economic empowerment and male engagement, can benefit nutrition outcomes (Madzorera and Fawzi 2020).

### 4.2 Food Environment Challenges and Proposed Solutions

Challenges in the *food environment*, including physical inaccessibility and perceived poor food safety and hygiene, were shared concerns across socio‐economic groups. These drivers have been previously identified in other Photovoice studies conducted in various Sub‐Saharan African countries (Liguori et al. 2022; Pradeilles et al. 2021; Trübswasser et al. 2021; Auma et al. 2020). Women in our study reported environmental sanitation and food adulteration as common issues in Ethiopia, motivating them to choose homemade foods for their children. Women also reported a willingness to use cow's milk as part of complementary food, however poor quality hindered its utilisation. A study in Ethiopia reported that milk adulteration was a common practice, with water added into milk along the dairy value chain, resulting in the consumption of poor‐quality and substandard milk (Zebib et al. 2023). Advertisement of unhealthy food products was identified as a key concern, but only among women of higher SES. They reported the use of appealing packaging and social media promotion of unhealthy foods, as well as the absence of healthy food promotion. This aligns with a narrative review on the digital marketing of unhealthy food and non‐alcoholic beverages, which reported that digital media exposure appears to influence the consumption of unhealthy foods (Fretes et al. 2025).

In response to these challenges, women suggested several strategies to improve the food environment. Women from higher SES groups proposed *agricultural actions*, including the promotion of community and home gardening. Combined with capacity‐building on garden development and counselling on healthy diets, these initiatives can increase the availability, accessibility, affordability and appeal of nutritious foods (Hawkes et al. 2020b; Ruel et al. 2018). Establishing markets to sell the surplus produce could also be a way to support income generation among urban poor women. Women also proposed *actions* linked to *supply chain infrastructure*, such as strengthening markets selling nutritious foods to low‐income communities and ensuring access to infrastructure to improve food safety (Hawkes et al. 2020b). Women also highlighted *business targeting initiatives*, such as proper monitoring and regulation of the foods sold by street vendors. Regulatory measures for informal food vendors should be paired with food safety awareness initiatives and supported by infrastructure and financial incentives to facilitate compliance. These efforts could be complemented by supporting vendors to include healthy food options in their menu in place of high sugar, fat and salt food options in and around work and schools, thereby improving the availability, access, affordability, and appeal of nutritious foods (Hawkes et al. 2020b). Regarding *regulations and laws*, women of higher SES proposed restricting unhealthy food advertisements. This could include limiting all forms of marketing, advertising, and in‐store promotions of unhealthy foods, particularly to children, which could help reduce the high appeal for these products (Boyland et al. 2025; Boyland et al. 2022; Hawkes et al. 2020b). In Ethiopia, although there are currently no specific regulations restricting unhealthy food advertising, the country's Excise Tax Proclamation (No. 1186/2020) imposes high taxes on imported unhealthy foods, such as sugar‐sweetened beverages and high‐fat products. However, implementing advertising and marketing restrictions would require stronger policy frameworks and enforcement mechanisms.

### 4.3 Economic Challenges and Proposed Solutions

In our study, *economic drivers* such as low income, unemployment and the high cost of healthy foods were consistently reported as key drivers of food choices, especially for participants from lower SES backgrounds. Photovoice studies in Ghana and Kenya documented similar results, highlighting that economic access and unemployment strongly influenced the types of foods consumers could access and purchase (Posey et al. 2024; Pradeilles et al. 2021). Research in India also identified price and affordability as key drivers of food acquisition patterns (Turner et al. 2022). In Ethiopia, from 2007 to 2016, the cost of all nutritious food groups, including grains, roots, and tubers; fruit and vegetables; pulses; and animal‐source foods, increased significantly, while the price of nutritionally‐poor food groups, such as oil, fat, and sugar, decreased (Ameye et al. 2021). Another study in Ethiopia found a 20% decline in the real food wage from 2020 to 2022 among urban workers (Headey et al. 2024). More recently, the national average cost of a healthy diet (i.e., the least expensive combination of locally available items that meets Ethiopia's Food‐Based Dietary Guidelines) rose by 7.0% between the third and fourth quarters of 2024 (Ethiopian Public Health Institute & National Information Platform for Nutrition 2024).

To address these economic barriers, women proposed a range of *financial* strategies. These included subsidising nutritious foods and implementing social safety net programmes, combined with nutrition education, to alleviate the economic burdens faced by urban women and improve diets. Evidence from previous studies indicates that such programmes can have positive impacts across multiple outcomes, including diets and nutritional status, however their long‐term scalability and sustainability remain uncertain and require further research (Ahmed et al. 2025; Gilligan et al. 2025; Hawkes et al. 2020b). Context‐specific financial actions, such as credit schemes (*Ekub*), were also proposed to mobilise and distribute resources within the community.

### 4.4 Strengths and Limitations

This study has several strengths. First, the use of the Photovoice methodology enabled participants to express their perceptions and challenges more effectively than conventional qualitative methods. The photographs, along with participants' narratives, served as a powerful advocacy tool to stimulate society‐led action (Auma et al. 2024). Additionally, focus group discussion workshops, rather than individual interviews, provided a supportive space to discuss, prioritise community problems, and collectively advocate for change. Second, we included women from both lower‐ and higher‐socio‐economic groups to capture a diverse range of perspectives. Third, while most research focuses on the underlying drivers of food choices and dietary behaviours, our study also explored proposed solutions proposed by women themselves, an essential step in developing context‐specific nutrition interventions. To our knowledge, this is the first study that allowed women to propose solutions to their own problems. One limitation of the study is that the perspectives of women in the selected neighbourhoods may not be representative of other communities in Addis Ababa. A second limitation is the relatively short engagement with women (3‐week period, two group sessions), which could not be extended due to participants' time constraints and the challenges of organising group sessions in urban areas. It would have been a good addition to organise a joint event bringing together women and decision‐makers but power dynamics, governance structures, and cultural norms made such interactions unfeasible. However, we held a workshop with diverse stakeholders from government, research and academia, the private sector, donors, and NGOs, presenting women's voices through a photography exhibition and booklet (Pradeilles et al. 2025). Stakeholders' perspectives and insights were gathered during this event.

## 5 Conclusion

This study aimed to explore the lived experiences of WRA and their children U5 in adopting healthy diets across lower‐ and higher‐SES groups in urban Ethiopia. The findings highlight that women recognised what interventions would work for them and the actors who should support them, underscoring the importance of including women's voices in decision‐making processes and institutionalising their participation in nutrition governance. The solutions proposed by women spanned multiple domains, sectors, and levels, reflecting women's understanding of the complex factors influencing dietary behaviours. Women from lower socio‐economic backgrounds mainly expected government‐led actions, whereas those from better‐off households also saw a role for society in driving change. Government‐level solutions included creating job opportunities, raising knowledge and awareness about healthy diets, improving access to nutritious and safe foods, expanding childcare services, and restricting the promotion and availability of unhealthy foods. Societal‐level solutions included promoting gender equality in caregiving, strengthening existing community‐based loan schemes, and supporting urban agriculture. Although the proposed solutions varied by SES, many could benefit all urban women and their families now or in the future. Implementing a range of integrated actions across the food system, targeting individual, food supply chain, food environment, and economic drivers, is essential for promoting healthy diets, thereby tackling multiple forms of malnutrition.

## Author Contributions

R.P., M.H., and M.R. designed the research study. R.P., M.W., M.H., M.R., K.B., M.S., and N.B. were involved in designing the data collection approach and tools. MW collected, transcribed, and translated the data. M.W., R.P., and A.I. analysed and synthesised the data. M.W. and R.P. wrote the first draft of the paper with critical input from M.H. and M.R. All authors reviewed the manuscript and approved the final version.

## Conflicts of Interest

The authors declare no conflicts of interest.

## Supporting information

Supporting File

## Data Availability

The data that support the findings of this study are available from the corresponding author upon reasonable request. The data supporting the findings of this study, including metadata and tools used for data collection and analysis, are published in the IRD's DataSuds repository (https://doi.org/10.23708/IWFBR4). The metadata will be fully publicly accessible upon publication of the associated article. The underlying data will be available upon request via the ‘Request access’ button on the DataSuds platform, after logging in with a CRU account or an account from a French university or research organisation.
