# Supplementary material for: Perceived Challenges and Solutions to Adopting Healthy Diets Among Women and Children: A Photovoice Study in Urban Ethiopia
Source: Matern Child Nutr. 2026 Jun 17;22(3):e70208. doi: 10.1111/mcn.70208 (PMC13273641; doi:10.1111/mcn.70208)
Supplement: Supplementary file 1 — Supporting File [file MCN-22-e70208-s001.docx]

**Supplementary materials**

**Supplementary material 1:** Conceptual framework for the TAMMIE project: the 4P cycle


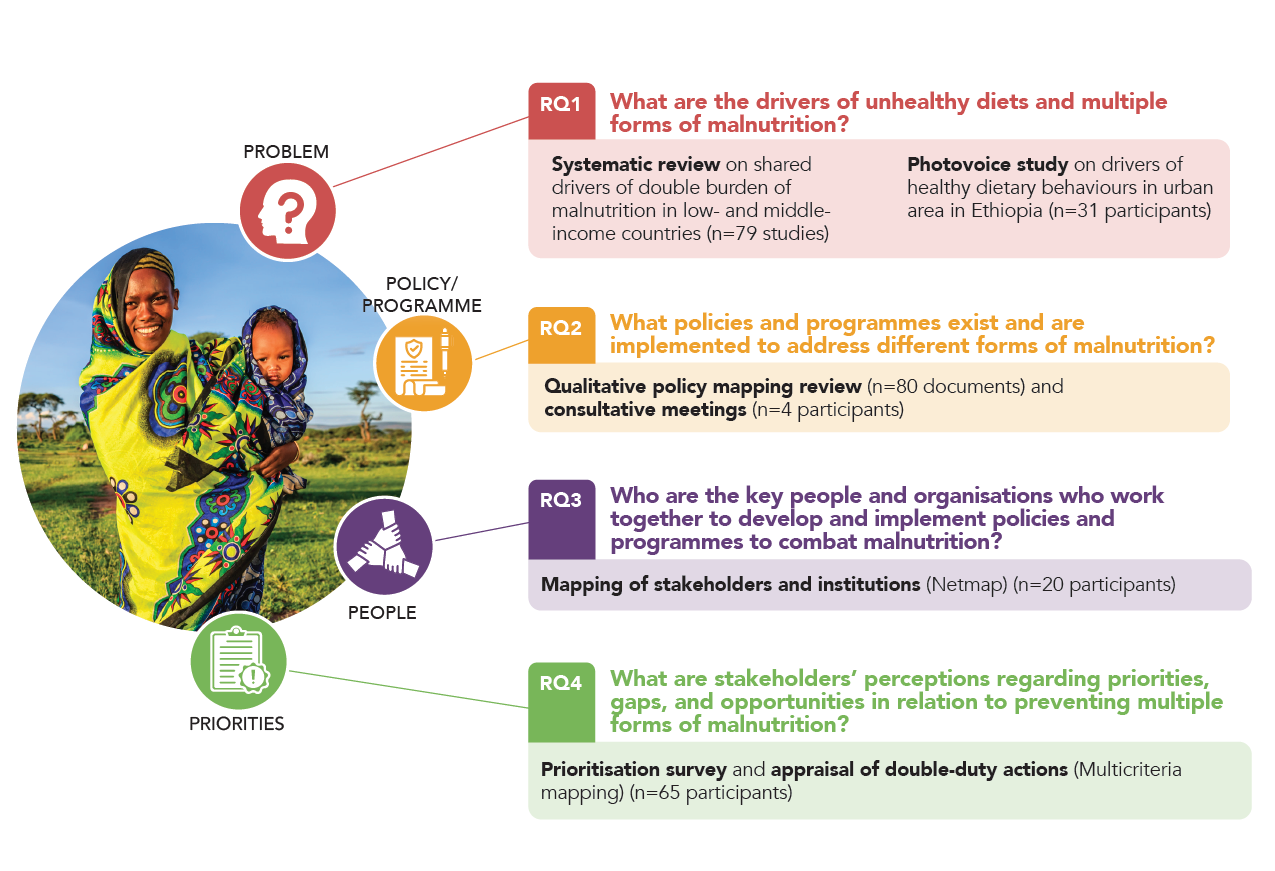


RQ: research questions

**Supplementary material 2:** Sampling and recruitment

|  | Not pregnant/lactating and not a mother | Pregnant | Lactating and mother of a child U2 | Not pregnant/lactating but mother of a child U5 | Total |
| --- | --- | --- | --- | --- | --- |
| Lower SES women (n=2 FGDs – style workshop) | n=4 | n=4 | n=4 | n=4 | n=16 |
| Higher SES women (n=2 FGDs – style workshop) | n=4 | n=4 | n=4 | n=4 | n=16 |
| Total | n=8 | n=8 | n=8 | n=8 | **n=32** |

**Supplementary material 3:** Interview guide for the introductory session of the Photovoice study

**Introductory session -** Presenting the photovoice exercise to participants

Good morning/afternoon, we are here to discuss the photovoice activity you will do as part of this study.

**What is Photovoice?**

A process of collecting information and expressing issues and concerns through photos. Photovoice asks participants to use photographs to record aspects of their lives and experiences.

In this project, we will be asking you to take some pictures of the things that have an impact on the healthiness of your diet and that of your children. We are doing this, so that we can help the formulation of policies that improve diets and nutrition in Ethiopia.

We will ask you to take pictures with your smartphones. The pictures you will take and the discussion we shall have with you on your pictures, will help us understand the challenges you are experiencing around healthy eating/feeding in your daily lives. We will also spend quite some time discussing solutions that would be appropriate to you to tackle those challenges.

To this end, we would like to share some of the photographs that you and the other women in this project take in a photography booklet so that people who make decisions to improve diets and nutrition can have a better idea of the things that affect you and the ways these could be solved. Your pictures can therefore be powerful in making change!

This Photovoice exercise, discussion of challenges for healthy dietary practices and context-appropriate solutions will take place in one full day workshop.

Today, we will take you through how to use the smartphone to take pictures. We will show you how to turn the camera on/off, how to snap the pictures and how to check if the pictures are on the phone or not. We will also show you how to use WhatsApp and how to transfer the pictures to the study team members.

There are some few things to note:

**Photo Ethics and Safety**

- If you take a picture and the face of person shows, you will need to ask permission from the person before the picture can be used.

- Though we want you to take these pictures for us, please ensure you are safe anytime you go out to take pictures.

- You can go with another person to take the pictures.

- Don’t do anything you wouldn’t usually do.

- Don’t go anywhere you wouldn’t usually go.

- Please do not enter into a person’s private space to take your pictures.

- Avoid disclosure of embarrassing facts about individuals through your pictures.

- Avoid taking pictures of people in such a way that leaves a negative and inaccurate impression about that person.

Let us start with showing you how to use the camera on your phone and after, we will discuss the theme we would like you to focus on. Please let us know if you do not understand or something is not clear. We will take you through it until everything is clear.

You will have 7 days to take pictures that depict the challenges you may face around eating healthily for you and your children. We will ask you to send us maximum four photographs and you will be able to do this via WhatsApp. We will then print your photographs and bring them to the workshop together. Your active participation and engagement is required.

We will provide you with a smartphone (deprived area only), internet, and airtime package for the duration of the study. You will get to keep the smartphone after the end of the study (deprived area only).

**Obtaining written consent from all participants**

We will now ask you to read the information sheet and consent form in Amharic and have you sign the consent form if you agree to participate in the study. Please read the information sheet for participants who cannot read and write.

If you have any questions, you can ask the facilitators.

(*Note: collect the written consent form from all participants when they finish and check whether it is signed properly or not. If it was not possible to get written consent, ensure to get verbal consent).*

**Fix date and place for the workshop**

Discuss and fix the date and place for the photovoice discussion, which is convenient for most participants (may be a week after sending the pictures).

**Supplementary Material 4:** **Interview protocol for the FGD**

- Print photographs (A5 size) for workshop

- Match photo numbers with participant number for data storage and identification on the day of interview.

- Conduct focus group discussions (see list of questions for the workshop below)

| **Participatory research steps** | **Questions** |
| --- | --- |
| FGD/workshop (with women)  (Morning session)- length 180 minutes | How would you define eating/feeding healthy for yourself?  How would you define eating/feeding your child healthily?  Please discuss the challenges you are facing around eating healthily for yourself and/or your children U5. You can use some of the pictures you took to help you talk through the challenges.  Together, can you please organize the different pictures you took in different themes/challenges?  Can you discuss the photographs and their meanings together?  Note for interviewer:  The group discussions will be guided using the PHOTO technique (could you talk about or describe your Photo; what is Happening in your photograph; why did you take a photograph Of this; what does this photograph Tell us and how can this photograph provide Opportunities for positive change).  Please agree on the top four challenges/themes that you are facing around eating healthily for yourself and or/ your children U5? Why did you choose these over other challenges?  Please select in group photographs that best depict those four challenges/themes (max 10-15) |
| FGD/workshop (with women) (Afternoon session)- 120 minutes | Please discuss solutions that you think would be appropriate to tackle the top four identified challenges? Why do you think these would work? What may be some of the barriers?  A four-point matrix will be used to guide the solutions discussions, in which the participants will indicate the **Idea** (the proposed solution), **Who should implement** (stakeholders responsible for implementing the solution), **How it should be implemented** (proposed steps/processes of implementing the solution) and **What is needed for implementing the solutions** (resources for implementation). |

**Supplementary Material 5**: List of identified challenges across socio-economic groups

| **Socio-economic group** | **Identified challenges** | **Prioritised challenges** |
| --- | --- | --- |
| **Lower SES** | Lack of awareness about a healthy diet | Lack of awareness about a healthy diet |
|  | Time constraints | Time constraints |
|  | Physical inaccessibility to healthy foods | Physical inaccessibility to healthy foods |
|  | Poor food safety and hygiene | Poor food safety and hygiene |
|  | Lack of cooking space and an unclean cooking area | Inadequate cooking space |
|  | Financial inaccessibility of healthy food | Financial inaccessibility of healthy food |
|  | Unequal food distribution among family members |  |
|  | Lack of modern kitchen materials |  |
|  | Shortage of transportation |  |
|  | Lack of household utility services |  |
|  | High cost of living |  |
| **Higher SES** | Time constraints | Time constraints |
|  | Preference for unhealthy food | Preference for unhealthy food |
|  | Physical (in)accessibility of (un)healthy food | Physical (in)accessibility of (un)healthy food |
|  | Poor food safety and hygiene | Poor food safety and hygiene |
|  | Unhealthy food promotion and advertisement | Unhealthy food promotion and advertisement |
|  | The nature of women’s jobs, such as frequent travelling |  |
|  | Unhealthy food menu in schools |  |
|  | Colleagues’ pressure on food choice in the workplace |  |
|  | Unavailability of pure and quality milk |  |
|  | Food choice during hormonal changes, such as menstruation and pregnancy |  |
|  | Poor road infrastructure |  |
|  | Availability of unhealthy foods |  |
|  | Poor sanitation and hygiene of street vendors |  |
|  | Unhealthy food at social events |  |
|  | Accessibility of unhealthy street food |  |
|  | High cost of food |  |
|  | Picky eating in children |  |
